# Two Girls With Adrenal Insufficiency and Failing Gonads

**DOI:** 10.1210/jcemcr/luad044

**Published:** 2023-05-08

**Authors:** Sarah Wing-Yiu Poon, Raymond Hang-Wun Li, Joanna Yuet-Ling Tung

**Affiliations:** Department of Paediatrics and Adolescent Medicine, Queen Mary Hospital, The University of Hong Kong, Hong Kong, China; Department of Obstetrics and Gynaecology, Queen Mary Hospital, The University of Hong Kong, Hong Kong, China; Department of Paediatrics, Hong Kong Children's Hospital, Hong Kong, China

**Keywords:** congenital lipoid adrenal hyperplasia, *StAR* gene, hypogonadism, adrenal insufficiency

## Abstract

Congenital lipoid adrenal hyperplasia (CLAH) is a rare cause of adrenal insufficiency caused by mutations in the steroidogenic acute regulatory (*StAR*) gene. Patients classically present with adrenal crisis in early infancy and female external genitalia irrespective of chromosomal sex. We report 2 Chinese patients with normal female external genitalia presenting with salt wasting in the neonatal period. However, the diagnosis of CLAH was made only during pubertal years when they developed hypergonadotropic hypogonadism. One of them was subsequently found to have a 46XY karyotype and gonadectomy was performed at age 15 years. The other patient developed gonadal insufficiency and polycystic ovaries after menarche with hemorrhage into ovarian cysts requiring cystectomy. These 2 cases illustrate the importance of recognizing atypical features in neonates presenting with adrenal crisis. In managing the newborn with adrenal insufficiency and female-appearing external genitalia, the possibility of sex reversal and diagnosis of CLAH should be considered. Accurate delineation of internal pelvic organs using reliable imaging modalities or even laparoscopy, together with careful interpretation of clinical and laboratory findings, are crucial to accurate diagnosis and subsequent management.

## Introduction

Congenital lipoid adrenal hyperplasia (CLAH) is the most severe form of congenital adrenal hyperplasia (CAH), characterized by impaired synthesis of all gonadal and adrenal steroid hormones. It is caused by mutations in the steroidogenic acute regulatory (*StAR*) gene and is inherited in an autosomal recessive mode [1, 2]. Affected individuals typically present with salt-wasting crisis in early infancy, and the adrenal glands are grossly enlarged with accumulation of cholesterol and cholesterol esters [2]. As Leydig cells are also destroyed early in gestation, resulting in impaired testosterone production, 46, XY infants with the classic form of CLAH are born with typical female external genitalia. On the other hand, 46, XX patients have normal female genitalia at birth and undergo spontaneous puberty, but present with anovulatory menstrual cycles and problems with fertility later in life [3].

We report 2 Chinese girls with CLAH presenting with adrenal insufficiency in the neonatal period. However, the diagnosis was made only during pubertal years when they presented with hypergonadotropic hypogonadism.

## Case Presentation

### Case 1

This patient was born full term in the 1990s with a birth weight of 3400 g. She was the first child of a nonconsanguineous Chinese couple. Physical examination at birth showed generalized hyperpigmentation and normal female external genitalia with no signs of virilization.

## Diagnostic Assessment

She remained well in the first few days of life. Investigations for hyperpigmentation on day 7 of life showed sodium 129 mmol/L, potassium 7.3 mmol/L, and metabolic acidosis with pH 7.24 and base excess −10.1 mmol/L. Paired urine sodium was 175 mmol/L and chloride was 163 mmol/L, suggestive of renal salt losing. There was no hypoglycemia or hypotension. Spot cortisol was low at 84 nmol/L (3 μg/dL) with no significant rise after synacthen stimulation (266 nmol/L at 30 and 249 nmol/L at 60 minutes) (9.6 μg/dL at 30 minutes and 9.0 μg/dL at 60 minutes). Paired adrenocorticotropin (ACTH) was elevated at 1635 pg/mL (360.0 pmol/L) (normal 9-52 pg/mL, 2.0-11.5 pmol/L). Plasma renin activity was greater than 20 ng/mL/hour (normal range, 0.68-1.36 ng/mL/hour recumbent) while plasma aldosterone was 900 pmol/L (32.4 ng/dL) (normal range for standing, 111-860 pmol/L [4.0 to −31.0 ng/dL], recumbent, 28-444 pmol/L [1.0-16.0 ng/dL]). 17-hydroxyprogesterone (17-OHP) was 0.6 nmol/L (2 ng/mL). Ultrasound scan showed enlarged adrenal glands with indistinct corticomedullary differentiation. She was started on hydrocortisone and fludrocortisone for adrenal insufficiency. Repeated ultrasound scan at 8 months of life showed normal-sized adrenal glands, and she was thriving with normal growth parameters.

At aged 12 years, she was noted to have no pubertal development and was later found to have primary gonadal failure (luteinizing hormone [LH] 22 IU/L and follicle-stimulating hormone [FSH] 31 IU/L, estradiol 33 pmol/L [9.0 pg/mL]). Ultrasound of the pelvis reported a very small uterus with both ovaries visualized, and the adrenal glands were noted as unremarkable. Karyotype subsequently came back to be 46, XY, with a repeated ultrasound of the pelvis showing similar findings. Laparoscopy was finally performed at age 15 years. Intraoperatively, no uterus was found and the gonads were normal in size with unremarkable pouch of Douglas. There was a 2.5-cm blind-ended vagina. Gonadectomy was performed and the pathology showed atrophic testes with no malignant changes. Testicular tissues with Sertoli cells and rare spermatogonia were seen within atrophic seminiferous tubules. Thick hyalinized basement membrane was observed around most of the tubules with no spermatogenesis seen. Epididymis and vas deferens were both present.

## Treatment, Outcome and Follow up

Molecular analysis later revealed homozygous nonsense mutation (Q258x) in the *StAR* gene, confirming the diagnosis of CLAH. The family was counseled on the diagnosis and the patient was started on estrogen replacement therapy.

## Case 2

A baby girl was born full term in the 1980s to a nonconsanguineous Chinese couple. There was an episode of hypoglycemia with jitteriness and a blood glucose of 1.1 mmol/L (19.8 mg/dL) in the first few hours of life, which was promptly treated with intravenous dextrose bolus. On the fourth day of life, hyponatremia (121 mmol/L) and hyperkalemia (5.7 mmol/L) were noted, which were corrected with intravenous fluid and sodium supplement.

She developed recurrent vomiting with poor weight gain since the third week of life. Physical examination showed generalized hyperpigmentation but otherwise normal female external genitalia. Investigations showed a sodium level of 122 mmol/L and potassium level of 8.4 mmol/L with elevated paired urine sodium. Serum cortisol was low, whereas ACTH and plasma renin activity were elevated. She was treated for primary adrenal insufficiency with hydrocortisone, fludrocortisone, and sodium supplement. The condition stabilized and she had normal growth and development.

The patient had breast development at age 11 years and menarche at age 13 years. Despite regular menstrual cycles initially, she developed oligomenorrhea 2 years after menarche.

## Diagnostic Assessment

Gonadotrophins were elevated (LH 39 IU/L and FSH 8 IU/L) with low estradiol (41 pmol/L) (11.2 pg/mL), while 17-OHP and testosterone were both undetectable. Ultrasound of the pelvis showed enlarged ovaries up to 39.4 mL on the right side and 25.8 mL on the left side with multiple ovarian cysts of varying sizes measuring up to 2 cm in both ovaries.

She was started on cyclic medroxyprogesterone for regular withdrawal bleeding. LH level subsequently dropped from 39 IU/L to 19 IU/L in 5 years. FSH remained at the level of 5 to 6 IU/L while estradiol rose to 137 pmol/L (37.3 pg/mL). Repeated ultrasound scan showed gradual reduction in ovarian volume to 10 mL. Molecular analysis finally revealed homozygous nonsense mutation (Q258X) in the *StAR* gene, confirming the diagnosis of CLAH.

She presented with acute abdominal pain and fever at age 37 years. Pelvic ultrasonography showed a 4 × 4 × 4 cm multiloculated right ovarian cyst and a 6 ×6 × 6 cm left ovarian cyst.

## Treatment, Outcome and Follow-up

An urgent bilateral ovarian cystectomy was performed, with histopathology suggestive of follicular and endometriotic cyst, respectively. She was continued on medroxyprogesterone, and serial imaging 1 year post operation showed recurrence of a cystic lesion in the right ovary. The option of cystectomy was offered but the patient opted for conservative management and surveillance by ultrasound for the time being.

## Discussion

We reported 2 Chinese girls with CLAH presenting with adrenal insufficiency and delayed puberty. CLAH is caused by autosomal recessive mutations in the *StAR* gene, which result in impaired cholesterol transport into the inner mitochondrial membrane and hence near absent or absent biosynthesis of all circulating adrenal and gonadal steroids [2, 4]. It is a rare condition in most populations, but more than 100 patients have been reported with more than 40 *StAR* mutations identified [2]. Although the clinical phenotype of CLAH was first described in the 1950s, pathogenesis and genetics of the disease were better understood only by the mid-1990s. It is now known that the pathophysiology of CLAH can be explained by the 2-hit model. The first hit is loss of *StAR*-mediated acute steroidogenic response as a result of the underlying mutation, with preservation of a low level of *StAR*-independent steroidogenesis. There is thus a compensatory increase in ACTH and LH levels, which causes accumulation of cholesterol and cholesterol esters in cells. This results in the second hit whereby the accumulated lipids and their autooxidation products damage cells and disrupt the *StAR*-independent steroidogenesis [2]. Because of a low index of suspicion of this rare form of CAH, and the lack of genetic tests at that time, the diagnosis of these 2 patients was made only in the mid-2000s when they reached puberty.

Retrospectively, there were several hints that could have led to earlier diagnosis. First, both girls presented with adrenal insufficiency in the neonatal period with normal female external genitalia. This is an unusual finding in the most common form of CAH caused by 21-hydroxylase deficiency, where excessive production of androgens leads to virilization of the female external genitalia [5]. This atypical finding should have prompted us to search for other causes accounting for the presentation.

The abnormally low or even undetectable 17-OHP and testosterone levels in our patients, together with glucocorticoid and mineralocorticoid deficiency, suggested disorders in the first step in steroidogenesis with impaired biosynthesis of all 3 classes of adrenal steroids (glucocorticoid, mineralocorticoid, and sex steroid). Apart from CLAH, *CYP11A1* mutations causing cholesterol side-chain cleavage enzyme (P450scc) deficiency and 3β-hydroxysteroid dehydrogenase 2 (3βHSD 2) deficiency also result in impaired production of glucocorticoid, mineralocorticoid, and sex steroid. Patients with deficient P450scc activity are clinically and biochemically indistinguishable from those with CLAH, but they typically lack the massively enlarged adrenals characteristic of CLAH [6]. As for 3βHSD 2 deficiency, the ratio of pregnenolone, 17 OH-pregnenolone, and DHEA (dehydroepiandrosterone) to their corresponding Δ4 compounds under ACTH stimulation is diagnostic. 17-OHP may also be elevated because of 3βHSD 1 activity [7]. On the contrary, all steroid hormones are low in CLAH. This illustrated the importance of being cautious with not just elevated intermediate steroids, but also abnormally low steroid levels. In the evaluation of patients with adrenal crisis, assessment of sex steroid production is of equal importance to that of glucocorticoid and mineralocorticoid.

The first patient was found to have enlarged adrenal glands initially. In fact, if further imaging, such as computed tomography or magnetic resonance imaging, was performed at that juncture, fat deposition in the adrenal cortex may have been identified and the diagnosis could have been made earlier. Similarly, accurate assessment of internal pelvic organs could provide more information on sex reversal. Limitation of ultrasound findings was demonstrated in our first case, where a repeated scan still showed “presence of uterus and ovaries,” and clinicians should recognize this in the evaluation and proceed to other imaging modalities or even laparoscopy when in doubt.

As illustrated in our cases, accurate diagnosis is essential to guide subsequent management, including gender assignment and gonadectomy. Undoubtedly, in this era of genetics and genomics, with the advent of whole-exome sequencing and whole-genome sequencing technologies, there have been growing insights into the genetics of adrenal insufficiency and associated molecular mechanisms, and the diagnosis can often be easily made with molecular testing. However, clinical correlation, based on clinical, biochemical, and radiological features, is still essential.

The phenomenon of multiple ovarian cysts with elevated LH/FSH ratio in our second patient has also been reported in other 46, XX girls affected by this disorder, although the underlying pathogenic mechanism has not been well studied [8-10]. With accumulation of lipids in follicular cells, estrogen synthesis is impaired. This results in a hypergonadotropic state, whereby the high FSH stimulates follicular growth while the blunted estrogen rise is not able to mount an LH surge to trigger ovulation despite a high basal LH level. The continual growth of the follicle with deficient ovulation may predispose to ovarian cyst formation. Hormonal replacement therapy has been reported to diminish the size of ovaries and ovarian cysts [8]. With risk of ovarian cyst complication, including torsion and hemorrhage, as observed in our patient, regular surveillance by pelvic ultrasonography and hormonal replacement therapy to correct the hypergonadotropic hypogonadism may thus be considered in girls with CLAH.

In conclusion, a diagnosis of CLAH should be considered when newborns present with salt loss and normal-appearing female genitalia. Accurate delineation of internal pelvic organs using reliable imaging modalities or laparoscopy, together with careful interpretation of biochemical parameters, are crucial in reaching an accurate diagnosis and directing subsequent management.

## Learning Points

- CLAH should be considered in newborns with adrenal insufficiency and normal-appearing female genitalia.
- Evaluation of glucocorticoid, mineralocorticoid, and sex steroid production is essential in one presenting with adrenal crisis.
- Limitations of ultrasound in the accurate delineation of internal pelvic organs should be recognized.
- Genetic analysis plays an increasingly important role in evaluation of adrenal insufficiency.
- Ovarian cysts and their associated complications should be monitored in CLAH.

## Contributors

All authors made individual contributions to authorship: S.W.Y.P. prepared the manuscript and performed literature review on the topic; R.H.W.L. offered expert advice on gynecological management of the 2 patients; and J.Y.L.T. was involved in the clinical management of the index cases and supervised the manuscript submission.

## Data Availability

Original data generated and analyzed during this study are included in this published article.

## Abbreviations

3βHSD: 3β-hydroxysteroid dehydrogenase
17-OHP: 17-hydroxyprogesterone
ACHT: adrenocorticotropin
CAH: congenital adrenal hyperplasia
CLAH: congenital lipoid adrenal hyperplasia
FSH: follicle-stimulating hormone
LH: luteinizing hormone
*StAR*: steroidogenic acute regulatory gene

## References

[1] Boettcher C, Flück CE. Rare forms of genetic steroidogenic defects affecting the gonads and adrenals. Best Pract Res Clin Endocrinol Metab. 2022;36(1):101593. 34711511 10.1016/j.beem.2021.101593

[2] Miller WL. Disorders in the initial steps of steroid hormone synthesis. J Steroid Biochem Mol Biol. 2017;165(Pt A):18‐37. 26960203 10.1016/j.jsbmb.2016.03.009

[3] Bose HS, Sato S, Aisenberg J, Shalev SA, Matsuo N, Miller WL. Mutations in the steroidogenic acute regulatory protein (StAR) in six patients with congenital lipoid adrenal hyperplasia. J Clin Endocrinol Metab. 2000;85(10):3636‐3639. 11061515 10.1210/jcem.85.10.6896

[4] Miller WL, Auchus RJ. The molecular biology, biochemistry, and physiology of human steroidogenesis and its disorders. Endocr Rev. 2011;32(1):81‐151. 21051590 10.1210/er.2010-0013 PMC3365799

[5] Claahsen-van der Grinten HL, Speiser PW, Ahmed SF, et al Congenital adrenal hyperplasia—current insights in pathophysiology, diagnostics, and management. Endocr Rev. 2022;43(1):91‐159. 33961029 10.1210/endrev/bnab016 PMC8755999

[6] Tee MK, Abramsohn M, Loewenthal N, et al Varied clinical presentations of seven patients with mutations in *CYP11A1* encoding the cholesterol side-chain cleavage enzyme, P450scc. J Clin Endocrinol Metab. 2013;98(2):713‐720. 23337730 10.1210/jc.2012-2828 PMC3565115

[7] Guran T, Kara C, Yildiz M, et al Revisiting classical 3β-hydroxysteroid dehydrogenase 2 deficiency: lessons from 31 pediatric cases. J Clin Endocrinol Metab. 2020;105(4):e1718‐e1728. 10.1210/clinem/dgaa022 31950145

[8] Shima M, Tanae A, Miki K, et al Mechanism for the development of ovarian cysts in patients with congenital lipoid adrenal hyperplasia. Eur J Endocrinol. 2000;142(3):274‐279. 10700722 10.1530/eje.0.1420274

[9] Jin HY, Choi JH, Lee BH, Kim GH, Kim HK, Yoo HW. Ovarian cyst torsion in a patient with congenital lipoid adrenal hyperplasia. Eur J Pediatr. 2011;170(4):535‐538. 21057961 10.1007/s00431-010-1342-0

[10] Tanae A, Katsumata N, Sato N, Horikawa R, Tanaka T. Genetic and endocrinological evaluations of three 46, XX patients with congenital lipoid adrenal hyperplasia previously reported as having presented spontaneous puberty. Endocr J. 2000;47(5):629‐634. 11200945 10.1507/endocrj.47.629

